# Regulation of Gastrointestinal Motility by Motilin and Ghrelin in Vertebrates

**DOI:** 10.3389/fendo.2019.00278

**Published:** 2019-05-17

**Authors:** Takio Kitazawa, Hiroyuki Kaiya

**Affiliations:** ^1^Comparative Animal Pharmacology, Department of Veterinary Science, Rakuno Gakuen University, Ebetsu, Japan; ^2^Department of Biochemistry, National Cerebral and Cardiovascular Center Research Institute, Suita, Japan

**Keywords:** energy homeostasis, ghrelin, motilin, gastrointestinal motility, vertebrates, evolution

## Abstract

The energy balance of vertebrates is regulated by the difference in energy input and energy expenditure. Generally, most vertebrates obtain their energy from nutrients of foods through the gastrointestinal (GI) tract. Therefore, food intake and following food digestion, including motility of the GI tract, secretion and absorption, are crucial physiological events for energy homeostasis. GI motility changes depending on feeding, and GI motility is divided into fasting (interdigestive) and postprandial (digestive) contraction patterns. GI motility is controlled by contractility of smooth muscles of the GI tract, extrinsic and intrinsic neurons (motor and sensory) and some hormones. In mammals, ghrelin (GHRL) and motilin (MLN) stimulate appetite and GI motility and contribute to the regulation of energy homeostasis. GHRL and MLN are produced in the mucosal layer of the stomach and upper small intestine, respectively. GHRL is a multifunctional peptide and is involved in glucose metabolism, endocrine/exocrine functions and cardiovascular and reproductive functions, in addition to feeding and GI motility in mammals. On the other hand, the action of MLN is restricted and species such as rodentia, including mice and rats, lack MLN peptide and its receptor. From a phylogenetic point of view, GHRL and its receptor GHS-R1a have been identified in various vertebrates, and their structural features and various physiological functions have been revealed. On the other hand, MLN or MLN-like peptide (MLN-LP) and its receptors have been found only in some fish, birds and mammals. Here, we review the actions of GHRL and MLN with a focus on contractility of the GI tract of species from fish to mammals.

## Introduction

Food intake, digestion of foods, and absorption of nutrients through the gastrointestinal (GI) tract wall are fundamental physiological events for living vertebrates. The GI system is the gateway for food entry, and it is well known that GI motility positively influences feeding behavior and contributes to the regulation of energy homeostasis. In general, GI motility of vertebrates is regulated by contractility of smooth muscles controlled by extrinsic parasympathetic and sympathetic neurons, intrinsic enteric sensory and motor neurons, and some GI hormones (1–3). Hormones are signal transduction molecules carried through the bloodstream to transmit biological information from one cell to another by activation of specific receptors on the target cells. Many GI hormones, including secretin, peptide YY, neurotensin, gastrin, gastrin-releasing peptide (GRP), cholecystokinin (CCK), somatostatin, ghrelin (GHRL), and motilin (MLN) have been identified. GI hormones are produced in specialized gut endocrine or enteroendcrine cells of the GI epithelium, and they act on other digestive organs, associated cells and vagal nerve afferent terminals. The GI tract has functions in controlling energy homeostasis through nutrient absorption and excretion, and several gut hormones have functional significance in the regulation of food intake, secretion of other hormones and control of GI motility (4–8).

MLN was identified in the 1970s (9, 10) and GHRL was identified in the 1990s (11). From their similarity in amino acid sequences of both ligands and receptors, it is thought that these two peptides originate from the same ancestral gene and form a peptide family (5, 12, 13). GHRL and MLN are mainly produced in the gastric and upper small intestinal (duodenum) mucosa, respectively, and they have some common functional characteristics such as regulation of GI motility and appetite, which have crucial roles for regulation of digestion and absorption of nutrients. Therefore, MLN and GHRL must be important GI peptides for energy homeostasis of living vertebrates (4, 5, 14, 15).

**Figure 1:**
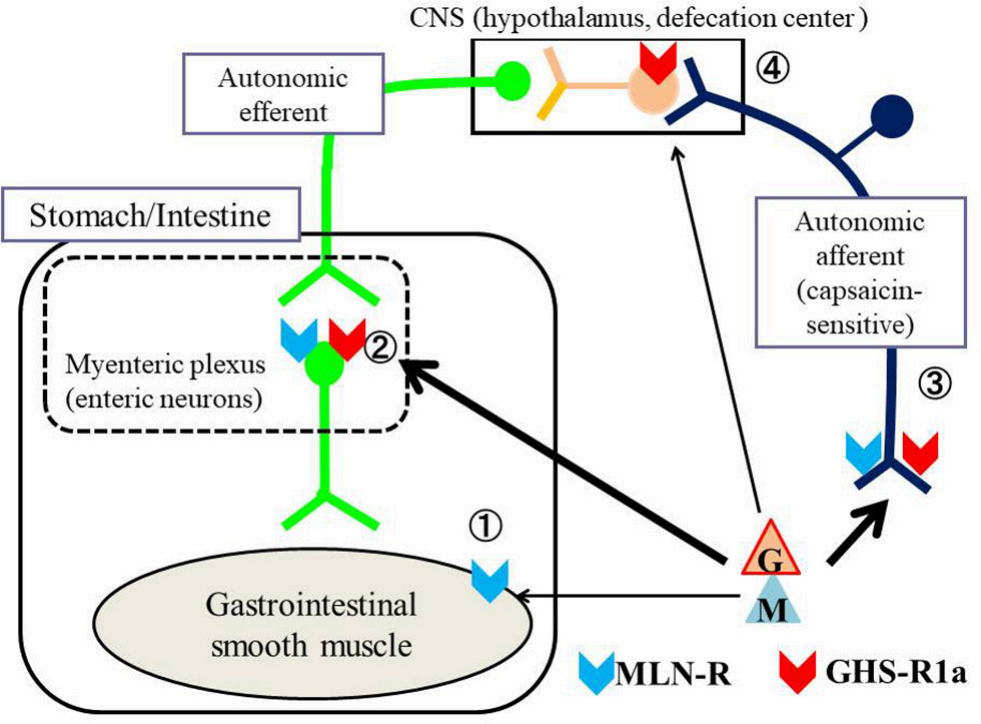
Possible action sites of MLN and GHRL for stimulating the gastrointestinal (GI) tract of vertebrates. The motility of the GI tract is regulated by efferent autonomic neurons and intrinsic neurons located in the myenteric plexus. MLN (M) and GHRL (G) affect the contractility of the GI tract through ① direct action on smooth muscle (receptors being present on smooth muscle cells), ② action on enteric neurons in the myenteric plexus (receptors being present on enteric neurons), ③ action on the nerve terminals of autonomic afferent neurons followed by excitation of the central nervous system (CNS), mainly the hypothalamus, which stimulates autonomic efferent neurons (receptors being present on afferent terminals) and ④ direct action on central neurons (receptors being present on neurons of the CNS). To produce a direct action on the CNS, it is necessary for MLN and/or GHRL-containing neurons to be present in the CNS or for both peptides that are produced in the gastrointestinal mucosa to be able to penetrate the blood-brain barrier. Locations of the motilin receptor (MLN-R, 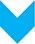 ) and ghrelin receptor (GHS-R1a, 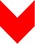 ) are also indicated in the figure. In general, MLN acts on receptors of smooth muscle, enteric neurons and afferent terminals of the vagus nerve, whereas ghrelin acts on receptors of enteric neurons, afferent terminals of the vagus nerve and central neurons.

In this review, we present results of recent studies regarding the regulation of GI motility by MLN and GHRL, with comparison to different vertebrates including mammals and non-mammals. Figure 1 shows the possible action sites of MLN and GHRL for stimulating GI motility in vertebrates: (i) smooth muscle cells, (ii) enteric neurons in the myenteric plexus, (iii) terminals of autonomic afferent nerves to evoke an afferent-efferent reflex such as a vago-vagal reflex, and (iv) direct action on the central nervous system (CNS). Generally, although it is possible in *in vivo* experiments on GI motility to detect the actions of peptides in all of the action sites indicated in Figure 1, in *in vitro* studies using isolated GI strips, only the actions on enteric neurons and smooth muscle cells can be detected.

## Regulation of Gastrointestinal Motility by MLN

**Figure 2:**
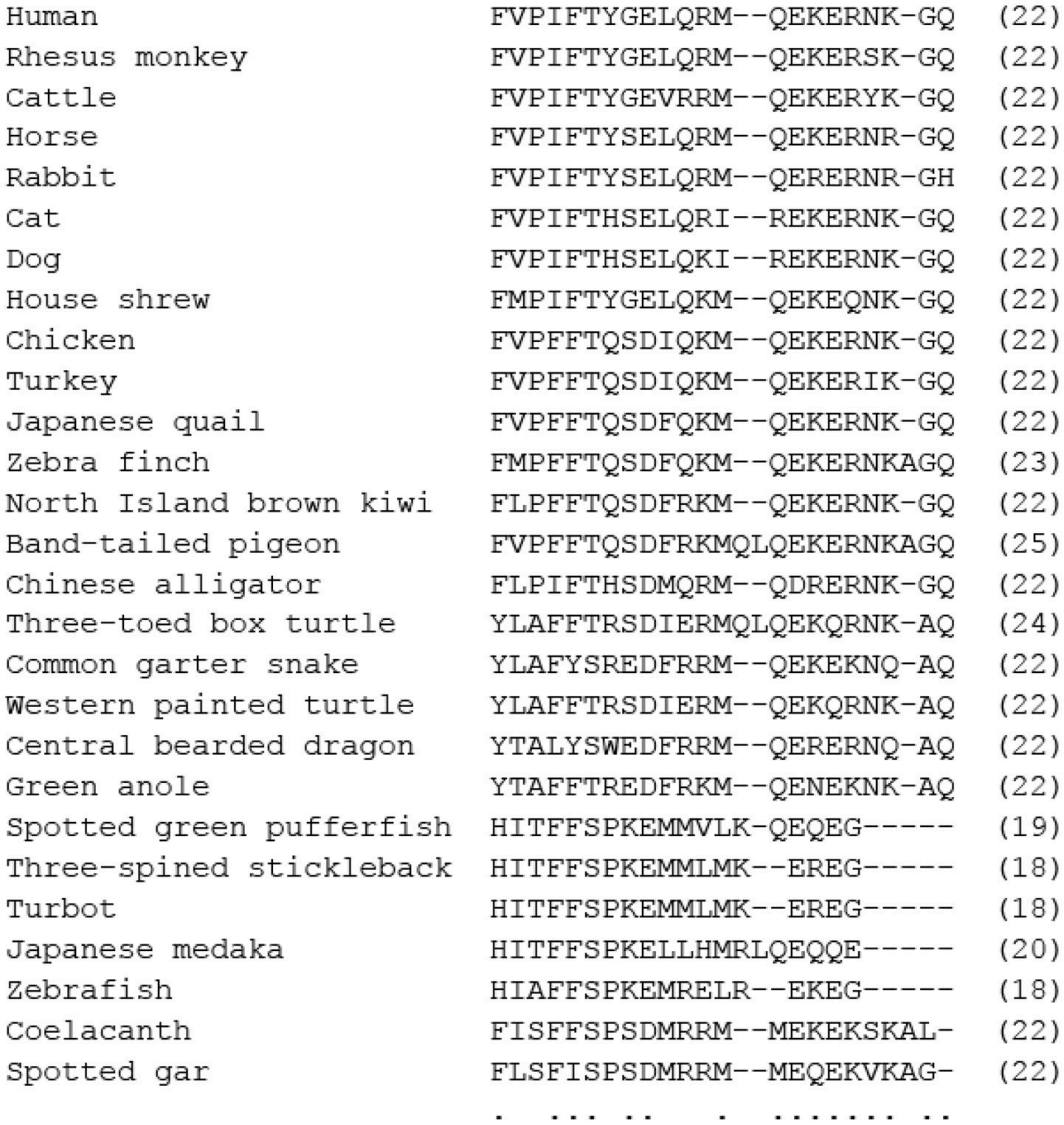
Comparison of amino acid sequences of mature motilin in vertebrates. Asterisks and dots indicate amino acids that are identical in all species or identical in more than half of the species. The number of amino acids is shown in parenthesis. Amino acid sequences were obtained from the DDBJ/EMBL/GenBank™ databases (acc#: NP_002409.1 for human, NP_001027979.1 for rhesus monkey, NP_776363.1 for cattle, NC_009163 for horse, X63860.1 for rabbit, NC_018727 for cat, XP_005627282.1 for dog, AB325968 for house shrew, NP_001292058.1 for chicken, XP_010722636.1 for turkey, BAU80773.1 for Japanese quail, XP_004175023.1 for zebra finch, XP_013812966.1 for North Island brown kiwi, OPJ88883.1 for band-tailed pigeon, XP_006025154.1 for Chinese alligator, XP_024054930.1 for three-toed box turtle, XP_013912065.1 for common garter snake, XP_005309417.1 for Western painted turtle, XP_020650577.1 for central bearded dragon, XP_008107992.1 for green anole, ALD51563 for spotted green pufferfish, ALD51564 for three-spined stickleback, AWP03197 for turbot, XP_023810781 for Japanese medaka, XP_002665930.1 for zebrafish, XP_005995529.1 for coelacanth, and NC_023181 for spotted gar).

MLN, a 22-amino-acid peptide hormone that was firstly discovered from the upper intestinal mucosa of a pig (9, 10) (Figure 2), is produced in enteroendocrine cells called M or Mo cells located in the mucosal epithelium in the upper small intestine, the duodenum. The name MLN originates from its stimulatory role in gut motility. Actually, MLN is known to contract the GI tract in several mammals through activation of smooth muscle cells, local enteric neurons and afferent terminals of vagus nerves (Figure 1). The mechanisms that have been identified depend on the experimental conditions (*in vitro* or *in vivo*) and animal species, such as dogs *(Canis lupus familiaris)*, rabbits (*Leporinae Trouessart*) and Asian house musk shrews (*Suncus murinus*) (4, 16–20). The MLN receptor (MLN-R) was identified in the human (*Homo sapiens*) stomach as an orphan G protein-coupled receptor (GPR38) for which the ligand is unknown, and it was deorphanized in 1999 (21). The MLN-R couples with G_q/11_ protein, stimulates phospholipase C that synthetizes inositol-trisphosphate, and increases intracellular Ca^2+^ and diacylglycerol to excite smooth muscles or neurons (22). *In vivo* and *in vitro* contraction studies for MLN have been extensively performed using GI tracts of various vertebrates in experiments.

### Mammals

#### Rat and Mouse

Mice (*Mus musculus*) and rats (*Rattus norvegicus*) are widely used as experimental animals in physiological and pharmacological studies. It has been known for a long time that MLN does not affect GI contractility *in vitro* (23, 24) and gastric emptying *in vivo* (25). Regarding *in vivo* recording of GI motility, interdigestive migrating contraction-like motility, with short intervals (about 15 min), was observed in the mouse and rat GI tracts in fasting periods (26, 27), but this motility pattern is actually caused by a family peptide, GHRL (see below). Recent genome-wide analysis revealed that these rodentia lack genes for MLN and its receptor (28, 29).

#### Guinea-Pig

The guinea-pig (*Cavia porcellus*) belongs to rodentia, as do rats and mice, but several findings indicate the possible presence of an MLN system. In a previous molecular biological study, an MLN precursor was identified in the duodenal mucosa (GenBank accession number AF323752) and the structure of guinea-pig MLN is estimated to be FVPIFTYSELRRTQEREQNKRL (30). The results of an immunohistological study, using a human MLN antibody and a human MLN-R antibody, suggested the presence of MLN and MLN-R protein in the GI tract (31). However, human MLN did not cause any contractions of the intestine either in a non-stimulated or electrically stimulated condition *in vitro* (23, 32). The guinea-pig MLN was also ineffective at contracting GI strips or at modifying neural responses in the guinea-pig (33) (Table 1). There has been no *in vivo* study in which the GI motility of the guinea-pig was recorded. In the guinea-pig MLN system, the MLN gene might be expressed but the MLN-R gene is degenerated, as in other rodent species such as the kangaroo rat (genus *Dipodomys*) (29, 33).

**Table 1:** Comparison of gastrointestinal motility-stimulating actions of motilin in several vertebrates.

**Species**	***In vitro*** **study**		***In vivo* study**	**Action site**	**References**
	**Unstimulated**	**Stimulated**			
**Fish**					
Rainbow trout		Potentiation of EFS			(34)
Zebrafish^*^	No effect				(35)
**Amphibians**					
Bullfrog	Contraction (human MLN)				(36)
Japanese fire belly newt	No effect (human MLN)				(36)
**Reptiles**					Research not available
**Birds**					
Chicken^*^			Contraction (rhythmic oscillatory complex)		(37)
	Contraction	Potentiation of EFS		Enteric neurons (proventriculus)	(38)
				Smooth muscle (proventriculus and ileum)	(39, 40)
Quail^*^	Contraction			Enteric neurons (proventriculus)	(41)
				Smooth muscle (proventriculus and ileum)	(41)
**Mammals**					
Human^*^	Contraction	Potentiation of EFS		Smooth muscle and enteric neurons	(42)
	Contraction			Smooth muscle	(43)
			Contraction (phase-III)		(44, 45)
Dog^*^	Contraction			Smooth muscle	(46)
			Contraction (phase-III)	Enteric neurons and vago-vagal reflex	(4, 17, 47–49)
Pig^*^	No effect	No effect (EFS)			(50)
Rat	No effect				(23, 25)
			No effect (gastric emptying)		(25)
Mouse	No effect				(24, 35)
Guinea-pig^*^	No effect	No effect (ganglion stimulant)			(23, 33)
		No effect (EFS)			(32)
Rabbit^*^	Contraction			Enteric neurons and smooth muscle	(16, 51–53)
			Contraction	Enteric neurons	(16)
Asian house musk shrew^*^	Contraction			Enteric neurons	(18, 19)
			Contraction (phase-III)	Enteric neurons	(20, 54)

* *Motilin structure has been identified in the marked animal species*.

*EFS, Electrical field stimulation for excitation of enteric neurons*.

#### Dog

Canine MLN is a 22-amino-acid peptide that has differences in five amino acids at positions of 7, 8, 12, 13, and 14 when compared with human MLN (Figure 2). Dogs have been used for a long time to examine the regulation of GI motility by autonomic nerves and gut hormones. The GI motility (circular muscle direction) of conscious dogs is measured by force transducers sutured on the serosal surface of the stomach and the intestine. The GI motility in dogs can be divided into two patterns, interdigestive and digestive (postprandial) contractions. GI contraction in the interdigestive state is called the interdigestive migrating motor complex (IMC), which is characterized by three different phases: phase-I (motor quiescent period), phase-II (irregular and low-amplitude contraction period) and phase-III (regular and high-amplitude contraction period) (4, 47, 49, 55). The IMC pattern in fasted periods has also been reported in the GI tracts of humans and house musk shrews (44, 54, 56). The physiological significance of the IMC is thought to be for flushing out and cleaning up the GI lumen, mechanically and chemically, to make it ready to receive the next food, and for preventing bacterial overgrowth in the intestinal lumen (4, 56, 57). MLN has been thought to be an endogenous regulator of phase-III activity of the IMC evoked in the stomach in a fasting state (47–49, 58). The following findings support the involvement of MLN in gastric phase-III: (i) the peaks of endogenous MLN change cyclically and the peaks of plasma MLN concentration are highly associated with gastric phase-III contractions, (ii) exogenously applied MLN causes phase-III-like gastric contraction, and (iii) phase-III contraction is disrupted by administration of anti-MLN serum or MLN-R antagonists such as MA-2029. MLN-induced gastric contractions in dogs were shown to be sensitive to a muscarinic receptor antagonist, atropine, and to a ganglion blocker, hexametonium, and it was shown that a neural pathway including acetylcholine (ACh) was stimulated by MLN and that vagus nerves have an important role in GI contraction induced by MLN *in vivo* (4, 17).

In an *in vivo* study using conscious dogs, erythromycin caused a phase-III-like contraction that is similar to the response of exogenous MLN in a fasted state (17, 55). This macrolide compound was shown to bind to the MLN-R of mammals (21, 51). From the words “motilin” and “macrolide,” macrolide antibiotics (including erythromycin) are called “motilides.” Motilides, which are erythromycin derivatives, have been known to induce GI contractions in humans (59, 60) and rabbits (61) as observed by MLN.

In comparison with many *in vivo* studies, little is known about the *in vitro* studies using isolated canine GI strips. Poitras et al. (46) showed that bath-applied canine MLN, but not human MLN, causes a contraction of canine duodenum muscle strips by direct action of MLN on muscle cells, which is different from results obtained in the *in vivo* studies.

#### Human

Human MLN is a 22-amino-acid peptide with the same structure as that of pig MLN (Figure 2). Similar to the GI motility pattern in dogs, GI motility in humans can be divided into interdigestive and digestive contractions. IMC-like motility is observed in the interdigestive state and its physiological significance is the same as that described in the section for dogs (44, 56). MLN is thought to be the mediator of the IMC elicited in the stomach because exogenous MLN causes an active front of the IMC and because plasma MLN concentration fluctuated in a cyclic manner with phase-III of the IMC being consistent with the peak of MLN concentration (44, 56, 62). Recently, it was proposed that the IMC in the GI tract signals hunger sensation from the periphery to the brain in humans. Therefore, MLN mediating the IMC is a hunger hormone in humans. Erythromycin derivatives (motilide) and MLN-R agonits also caused phase-III contraction and hunger sensation in humans (59, 60). Due to their GI motility stimulation action and their resistance to degradation in a stomach, clinical use of erythromycin derivatives as gastroprokinetic agents is being investigated (63).

*In vitro* mechanical studies show that MLN causes contraction of human GI muscle strips. Ludtke et al. (43) reported the region-dependent direct actions of MLN on GI smooth muscle strips. Broad et al. (42) demonstrated both indirect action through activation of enteric neurons and direct action on smooth muscles with electrically stimulated GI strips. Low concentrations of MLN act on neural MLN-R, whereas high concentrations of MLN act on smooth muscle MLN-R (42). Therefore, it is thought that neural MLN-R might be function for regulation of GI contractility.

#### Rabbit

Rabbit MLN is also a 22-amino-acid peptide hormone with the structure of FVPIFTYSELQRMQERERNRGH, and with 5 amino acids at positions 8, 16, 19, 20, and 21 being different from those of human MLN (Figure 2). An immunohistochemical study indicated that MLN-producing cells were localized preferentially in the mucosa of the upper small intestine (duodenum), as has been reported in other species (64).

An *in vivo* study, in which myoelectric activity of the GI tract was recorded in conscious rabbits, indicates that the migrating myoelectric complex (MMC) originates from the proximal jejunum, not the stomach, and that the MMC appears both in feeding and fasted rabbits at almost the same intervals (65). Therefore, the character of the rabbit MMC is different from that of the IMC observed in a fasted state of dogs and humans. The effects of MLN on rabbit GI motility have been examined in an *in vivo* experiment under an anesthetized condition, and it was found that MLN caused contractions of the stomach and colon but not ileum. Atropine significantly decreased the contraction in the stomach but not in the colon, indicating that different contractile mechanisms are present between stomach and colon (16). The GI region-dependent motility stimulation action of MLN has also been demonstrated in an *in vitro* study using different parts of isolated muscle strips. Pharmacological analysis using atropine and tetrodotoxin (a neuronal blocker) indicated that MLN acted on smooth muscle MLN-R and in addition to the neural MLN-R located on cholinergic nerves (52, 53). The magnitude of MLN-induced contraction differed depending on the GI region (duodenum = jejunum > colon > stomach > ileum), probably due to different expression levels of the MLN-R, although the expression pattern has not been examined (16). High sensitivity of the colon to MLN is a characteristic of the rabbit GI tract. Rabbits belong to lagomorpha, not rodentia, and are coprophagous grass-eating animals with a property of hindgut fermentation. Therefore, regulation of colonic motility must be important, and in this situation, MLN might play a physiological role in the regulation. Regarding regulation of the rabbit MMC described previously, although the jejunum, the starting region of the MMC, is highly sensitive to MLN, the physiological role of MLN in regulation of the MMC has not yet been clarified.

#### House Musk Shrew

The effects of MLN on GI motility have been mainly investigated using dogs (*in vivo*) and rabbits (*in vivo, in vitro*) as mentioned earlier. However, since the body sizes of these animals are relatively large. In this point, house musk shrew (*Suncus murinus*) belongs to insectivora, different from mice, rats and guinea-pigs. The body size is similar to that of rats, and is easy to handle. Interestingly, because house musk shrew has both MLN and GHRL (66, 67), it is a good animal model to investigate their actions and interactions.

A molecular study revealed the primary amino acid sequence of the house musk shrew MLN (FMPIFTYGELQKMQEKEQNKGQ), and showed that three amino acids at positions 2, 12 and 18 were different from those of human MLN [(67), Figure 2]. MLN-producing cells localizes in the mucosa of the upper small intestine (duodenum) as in other mammals (67).

An *in vivo* study in which GI motility was recorded using conscious house musk shrews indicated that the GI motility pattern differs depending on the feeding conditions, and both interdigestive (IMC) and digestive motor patterns, that are similar with humans and dogs, were observed (54). Although changes in the plasma MLN concentration during the three phases of the IMC have not been examined, MLN caused phase-III activity of the IMC, and the phase-III activity was inhibited by a MLN-R antagonist (19, 54). These results suggest that MLN is involved in the induction of phase-III of the IMC as observed in humans and dogs. A functional role of the vagus nerves for regulation of MLN-induced contraction was demonstrated in a digestive state but not in the interdigestive state. MLN does not cause contraction in the digestive state in the vagus nerve-intact animals but causes contraction in vagotomized animals, suggesting that the vagus nerve suppresses the action of MLN in the digestive state (20). MLN also causes contraction of gastric strips in an *in vitro* study, and the contraction was completely abolished by atropine and tetrodotoxin, indicating that MLN-induced response is a pure neural origin (18). Namely, the MLN-R is located only in enteric neurons in the house musk shrew and mediates the MLN-induced contraction (18). In this animal species, interaction of MLN and GHRL for the regulation of GI motility was examined in both *in vivo* and *in vitro* studies [(19, 20, 68), see below].

#### Pig

Pigs (*Sus scrofa domesticus)* are the species in which MLN was first identified (9, 10), and the amino acid sequence of MLN is the same as that of human (Figure 2). An immunohistochemical study indicated that MLN is co-localized and secreted together with GHRL from the endocrine cells of the pig small intestine (69). However, the effect of MLN on regulation of GI motility has not yet been examined *in vivo*. Only an *in vitro* study has indicated that MLN does not cause contraction of GI strips and does not modify neural responses in the stomach and the intestine (50).

### Birds

#### Chicken

The morphology and function of the GI tract in birds are different from those in mammals in the following three aspects: (i) the crop in the middle of the esophagus stocks food, (ii) there are two distinct stomach structures, the proventriculus that secretes digestive enzymes for chemical digestion and the gizzard for mechanical digestion, and (iii) a pair of a long cecum and short colon/rectum. Electromyogram measurements showed that the MMC, consisting of three phases like the mammalian IMC, is also present in the GI tract of avian species including chickens (*Gallus gallus domesticus*) (70, 71). However, the MMC originates from the duodenum, not the stomach, and it appears in both fed and fasted states, being different from the mammalian IMC. The detailed mechanisms of regulation of the MMC in chickens have not been reported yet, but it is known that the appearance of the MMC is modulated by some gut hormones such as CCK and gastrin. The difference in the motility of the GI tract in fed and fasted states is unclear at present in the case of the bird MMC (70, 71).

In birds, MLN was first identified as a 22-amino-acid peptide in the extract of chicken duodenal mucosa (38). Six amino acids are different from those of human MLN at positions 4, 7–10, and 12 (Figure 2). The primary structure of MLN has been identified in several birds including the quail (*Coturnix japonica*), turkey (*Meleagris gallopavo*) and pigeon (*Columbidae*), and the structural difference in MLN among birds is small compared with that in mammals (Figure 2). Chicken MLN is produced in the mucosa of the duodenum but not in the proventriculus and gizzard, as in mammals (38). The chicken MLN-R has also been identified, and the homology to the human MLN-R was shown to be 59.1% (72). This value is lower than the homology to the MLN-R of other mammals such as the rabbit (84%), house musk shrew (76%), and dog (71%) (73–75), suggesting a different structure of the chicken MLN-R from that of mammals. The insensitivity to a mammalian MLN-R agonist, erythromycin, and low sensitivity of human MLN in the chicken intestine (38, 40) support the possibility of a structural difference of the chicken MLN-R from the mammalian one.

The GI-stimulating action of MLN has been mainly investigated using isolated smooth muscle strips *in vitro*, and it has been shown that MLN causes contraction of the proventriculus and small intestine but that the crop and colon are insensitive. The underlying mechanisms of MLN-induced contraction in the proventriculus are myogenic and neural mechanisms, being different from those in the small intestine only by myogenic action (40) (Table 1). The small intestine is highly sensitive to MLN, independent of aging due to a high expression level of MLN-R mRNA (76), but the physiological roles of MLN for regulation of GI motility in avian species have not been fully elucidated. Jimenez et al. (70) found rhythmic oscillating contraction in the chicken ileum, and Rodriguez-Sinovas et al. (37) reported that the plasma MLN concentration was high during spontaneous rhythmic oscillating contraction and that exogenous MLN triggered the contraction. These results suggested the involvement of MLN in regulation of the MMC in the small intestine. The proventriculus also shows an appropriate sensitiveness to MLN, but MLN-R mRNA expression significantly decreases with aging, and MLN-induced contraction also decreases markedly with aging (72, 76). This suggests the age-dependent regulation of proventriculus motility by MLN in adult chickens.

#### Japanese Quail

MLN has also been identified in the Japanese quail. Quail MLN consists of 22-amino-acids and only one amino acid at position 10 is different from that in chickens [(41), Figure 2]. The distribution of MLN-immunopositive cells in the quail is almost the same as that in the chicken (38). The effect of MLN on the quail GI tract is only examined in *in vitro* using isolated muscle strips. Chicken MLN is capable of causing contraction of the quail GI tract. The region-related contractile responsiveness (small intestine > proventriculus > crop = colon) and region-related different contraction mechanisms reported by Apu et al. (41) are similar to those in chickens (39, 40, 77) (Table 1). Although it is necessary to examine the responses of MLN in other avian species, the characteristics of the MLN-induced GI contraction in the two species are almost the same, and the high sensitivity of MLN in the small intestine suggests that MLN might regulate motility of the small intestine by spontaneous oscillating contraction as seen in chickens (37).

### Reptiles

Little is known about the MLN system in reptiles. An immunohistochemical study using an anti-human MLN antibody failed to show the presence of MLN in the digestive tract of three reptiles (*Testudo graeca, Mauremys caspica*, and *Lacerta lepida*), though the presence of other gut peptides, including neurotensin, gastrin, glucagon, and somatostatin, was demonstrated (78). On the other hand, MLN immunoreactivity was detected in the alimentary tract of the King's skink (*Egernia kingii*) (79). The presence of an MLN peptide possessing a mammalian MLN structure is dependent on the species of reptiles, and reptiles might therefore be the boundary for the presence of a mammalian-like MLN structure. Concerning the MLN structure, Liu et al. (80) reported that the deduced amino acid sequence of the lizard MLN was YTAFFTREDFRKMQENEKNKAQ, which is quite different from the N-terminal structure of human MLN (FVPIFTYGEL) and chicken MLN (FVPFFTQSDI). Figure 2 also indicates the different N-terminal structures of reptile MLNs from those of birds and mammals. However, the N-terminal structure of alligator MLN (FLPIFTHSDM) is close to that of the chicken.

Concerning the contraction study, the effects of human MLN have never been examined despite the fact that GI motility of isolated strips has been investigated in reptiles (81). Identification of MLN peptide and examination of its effects on reptile GI motility should be carried out in the future.

### Amphibians

No evidence has been obtained for the immunohistochemical localization and the structural difference of MLN in amphibians.

Previous studies using the isolated spinal cord of a toad (*Bufo marinus*) indicated that human MLN depolarized the motor-neurons of hemisected spinal cord *in vitro* (82). Our previous study *in vitro* using the isolated GI tract of the bullfrog (*Lithobates catesbeiana*) showed that human MLN induced small contractions of longitudinal muscle strips of the upper intestine at a relatively high concentration (1 μM) (36), while longitudinal muscle strips from the middle and lower intestine and gastric longitudinal and circular muscle strips were quite insensitive (Table 1). These results indicate that the upper intestinal strip, corresponding to the duodenum, was sensitive to human MLN. In mammalian and avian species, the GI region-related contraction of MLN, probably due to the heterogeneous expression of MLN-R, is one of the characteristics of the MLN-induced contraction in the GI tract (16, 39, 40, 76). The characteristic of region-dependent sensitivity might be preserved in the bullfrog GI tract. The above described results suggest the presence of MLN-R in the bullfrog GI tract. However, preliminary data indicated that erythromycin did not cause any contraction of the upper intestine of the bullfrog, suggesting the possibility that the structure of MLN-R is different from that in mammals. In contrast to the bullfrog, human MLN did not induce any contraction of the upper small intestine from another amphibian, the Japanese fire belly newt (*Cynops pyrrhogaster*) (36) (Table 1). Amphibians are divided into three groups, anuran, urodelal, and gymnophional species. The results suggest species differences in response to MLN among evolutional states of amphibians, similar to that for rodentia, and even in mammals that do not have a MLN system.

### Teleost Fish

The presence of a MLN peptide in the GI tract of a teleost was first investigated immunohistochemically by Pan and Fang (83), who reported that no MLN-immunoreactive cells were detected in the gut of a grass carp (*Ctenopharyngodon idellus*) (stomach-less fish) by using an anti-human MLN antibody. However, a recent molecular cloning study for MLN and MLN-R genes indicated the presence of MLN-like peptide (MLN-LP) and MLN-R genes in the zebrafish (*Danio rerio*) (80). The genomic structure was similar to that of the human MLN and GHRL genes (84, 85): The MLN-LP genes contain five exons and four introns, and the mature peptide was encoded by the second and third exons, although the amino acid sequence of the mature peptide is quite different from that of the known MLN, suggesting that the MLN-LP is unique in teleost (80) (Figure 2). The well-known MLN is composed of a 22-amino-acid sequence in both mammalian and avian species, but the structure of zebrafish MLN-LP is unique (80) (Figure 2). The N-terminal structure (FVPIFT) is essential for biological activity of MLN (86, 87), but the N-terminal structure (HIAFFS) of the zebrafish MLN-LP is quite different. The MLN-LP gene has also been identified in other fish, and the deduced amino acid sequences were HITFFSPKEMMVLKEQE (17 amino acids) in Takifugu (*Takifugu rublipes*) and HITFFSPKELLHMRLQEQQE (20 amino acids) in Medaka (*Oryzias latipes*) and these MLN-LPs are considered to be orthologs of the known MLN (80) (Figure 2).

In zebrafish, the MLN-LP gene was highly expressed in the intestine, moderately expressed in the liver, and expressed at low levels in the brain, heart and kidney (80). Interestingly, high expression of the MLN-LP gene in the liver has not been reported in mammals and birds. The ortholog of the MLN-R gene has been identified in zebrafish (34), and mammalian cells transfected with this receptor mRNA responded to the newly identified zebrafish MLN-LP (35, 80). These results indicate that an MLN system related to MLN-LP is present in zebrafish.

GI motility stimulation actions of MLN is mainly investigated *in vitro*. In isolated intestinal bulb and mid/distal intestine preparations of zebrafish, human MLN caused contraction of the intestinal smooth muscle. Erythromycin also induced contraction (34), though this compound was insensitive in the chicken GI tract (40). Interestingly, zebrafish MLN-LP caused contraction of the rabbit duodenum, but the affinity and efficacy were smaller than those of human MLN, indicating that zebrafish MLN-LP is a partial MLN-R agonist for mammalian MLN-R, although there is a substantial sequence difference (35). In the zebrafish intestinal bulb, zebrafish MLN-LP and human MLN induced only very small contractions at high concentrations (1–10 μM), although ACh caused marked contraction at a concentration of 1 nM−1 μM (Table 1). Liu et al. (80) examined the expression of MLN-R mRNA in zebrafish and reported that the expression level was highest in the brain (especially in the hypothalamus) and low in the intestine. This might explain the low responsiveness of MLN-LP in zebrafish and suggests that the MLN system is not positively involved in the regulation of intestinal motility in zebrafish (35).

On the other hand, high expression of the MLN-R gene in the hypothalamus and hindbrain suggests functions of MLN in the CNS other than GI motility in zebrafish, such as food intake and energy homeostasis (80). In mammals, similarly, MLN-binding sites (MLN-R) in rabbit cerebellum cells (88) and human cerebellum cells (89) have been reported. However, our data indicated that the expression level of MLN-R genes was low in the zebrafish brain, similar with that in GI tracts (35). Detailed experiments are needed to clarify the presence of MLN-R in the CNS of zebrafish. Taken together, the results indicate that MLN-LP is expressed in the GI tract of fish but that its physiological role for regulating GI motility is skeptical due to low expression of the MLN-R. Expression of the MLN-R in the CNS suggests a novel function of MLN in fish.

## Regulation of Gastrointestinal Motility by GHRL

GHRL, a gut peptide consisting of 28 amino acids, was identified from rat stomach extracts as an endogenous ligand of a G protein coupled receptor (GPCR), growth hormone secretagogue receptor 1a (GHS-R1a), and it was shown to primarily stimulate the release of growth hormone (GH) from a pituitary (11, 14, 90). GHRL has a unique structural feature with fatty acid modification at the third serine residue (Ser-3), and acylation is essential for binding of GHRL to GHS-R1a and for eliciting its biological actions (11, 14). GHRL is mainly produced in cells called X/A-like endocorine cells in the mucosa of the stomach as an unacylated-type, and Ser-3 is modified by a middle-chain fatty acid such as *n*-octanoic acid or *n*-decanoic acid through GHRL-*O*-acetyltransferase (GOAT) that co-localizes in the same X/A-like cells (91). A relatively high concentration of unacylated GHRL is present in the stomach and plasma, but its physiological roles have not been fully clarified (14, 92). Regarding the sites of action (receptors), two types of GHS-R, designated type 1a (GHS-R1a) and type 1b (GHS-R1b), have been found, and GHS-R1a with seven transmembrane domains is activated by GHRL, whereas GHS-R1b with five transmembrane domains is not (14, 90, 93, 94).

GHRL was first discovered as an endogenous GH secretagogue, but subsequent studies indicated that GHRL is also produced in the hypothalamic arcuate nucleus and is involved in the regulation of food intake through GHS-R1a expressed on neuropeptide Y and orexin neurons (95–97). Consistent with its effect on feeding, plasma GHRL concentration increases after fasting, and the increased concentration of GHRL stimulates food intake (12, 14, 98). On the other hand, it has been shown that GHS-R1a mRNA and protein is expressed in regions of the CNS other than the hypothalamus, such as the hippocampus, substantia nigra, ventral tegmental area and dorsal and medial raphe, as well as in various peripheral organs including the stomach, intestine, pancreas, thyroid, adrenal gland, kidney, heart and blood vessels (99–102). This ubiquitous expression of GHS-R1a mRNA and its protein suggests that GHRL has multiple functions, and accumulating evidence indicates its involvement in glucose metabolism, lipid metabolism, endocrine/exocrine functions, GI motility, cardiovascular functions, and reproduction (14, 84, 90).

**Figure 3:**
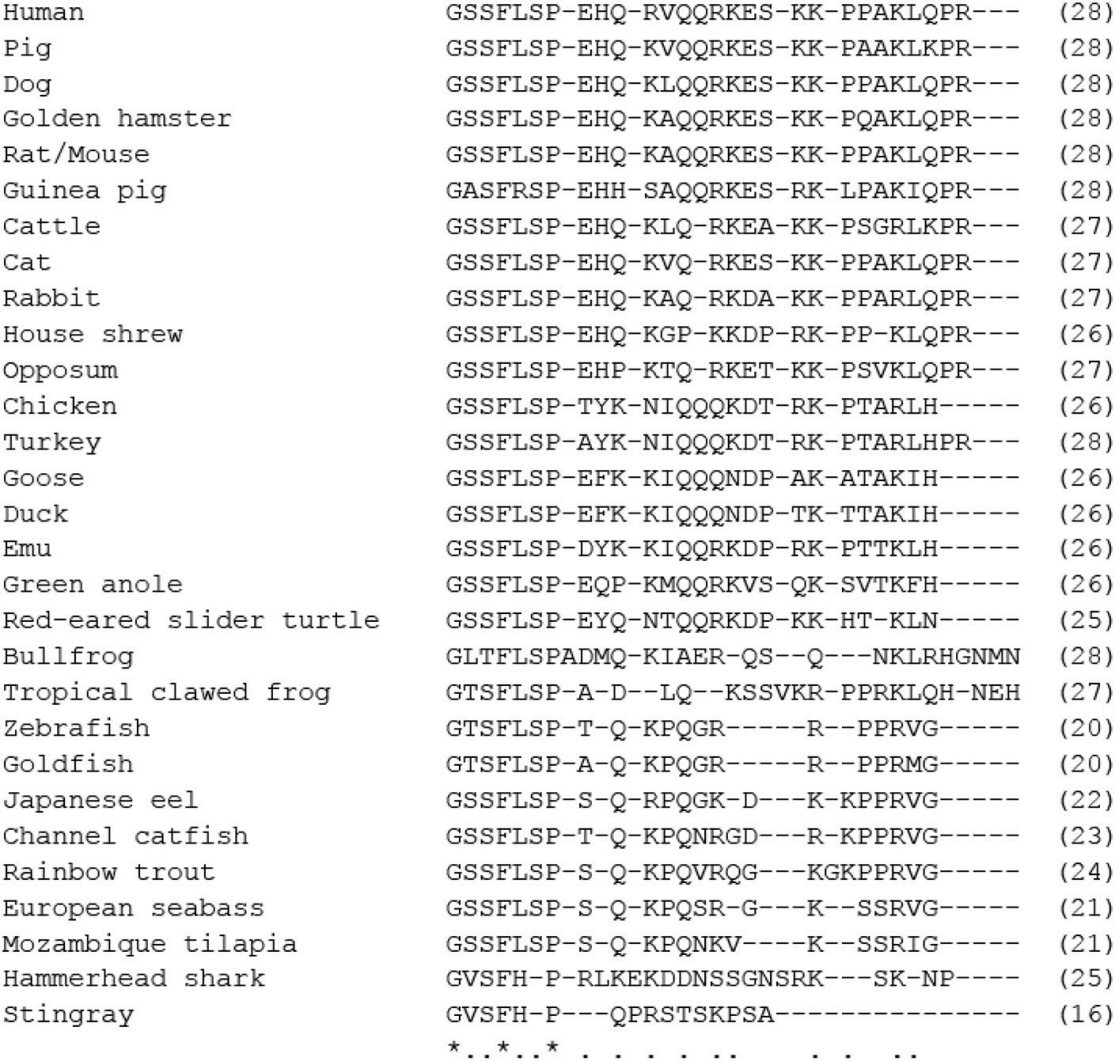
Comparison of amino acid sequences of mature ghrelin in vertebrates. Asterisks and dots indicate amino acids identical in all species or identical in more than half of the species. The number of amino acids is indicated in parenthesis. Amino acid sequences were obtained from the DDBJ/EMBL/GenBank™ databases (acc#: AB029434 for human, AY028942 for pig, AB060700 for dog, NM_012488 for mouse, NM_021669 for rat, NC_037349 for cattle, AB089201 for cat, XM_002722463 for rabbit, AB364508 for house shrew, XM_001375640 for opossum, AB075215 for chicken, XM_003210209 for turkey, AY338465 for goose, EF613551 for duck, AY338467 for emu, NW_003338820 for green anole, AB161457 for red-eared slider turtle, AB058510 for bullfrog, NC_030680 for tropical clawed frog, NM_001083872 for zebrafish, AF454389 for goldfish, AB062427 for Japanese eel, AB196449 for channel catfish, AB096919 for rainbow trout, DQ665912 for European seabass, AB077764 for Mozambique tilapia, AB254128 for hammerhead shark, and AB4800033 for the red stingray).

GHRL has been identified not only in mammals but also in non-mammalian vertebrates from elasmobranch fish to birds (84, 103) (Figure 3). Evidence indicates that GHRL is predominantly produced in the stomach of all species with stomachs and that it is mainly produced in the intestine in stomach-less animals such as goldfish. The fundamental structure of GHRL, such as a common sequence at the N-terminal seven amino acids with an acyl modification at Ser-3 (Thr-3 in the bullfrog), has been conserved during vertebrate evolution, with some exceptions in fish and amphibians (Figure 3). Phylogenetic tree analysis indicated that GHRL falls into two lineages, mammalian-type and cartilaginous fish-type (103), and it was very recently proved that the relationship between the two is not olthologous but paralogous (13). GHRL receptors have also been identified in non-mammalian vertebrates and have been roughly divided into two groups, GHS-R1a and GHRL receptor-like receptor (GHS-R1a-LR) (84, 103–105). Results of studies on the effects of GHRL in vertebrates have suggested that the GH-releasing action is a common action of GHRL (106–110).

From the genetic structural features with strong similarity in peptide precursor and receptor levels, GHRL and MLN originate from a common ancestral gene and form a family (13): GHS-R1a and MLN-R belong to a family including G protein-coupled receptor (GPR) 39, neurotensin and neuromedin-U receptors (111) and show 52% overall amino acid identity and 86% identity in the seven-transmembrane regions (5, 12). As mentioned earlier, MLN regulates GI motility in several mammals, and GHRL is known to regulate GI function in mammals and non-mammalian vertebrates (Table 2).

**Table 2:** Comparison of gastrointestinal motility-stimulating actions of ghrelin in several vertebrates.

**Species**	***In vitro*** **study**		***In vivo* study**	**Action site**	**Reference**
	**Unstimulated**	**Stimulated**			
**Fish**					
Rainbow trout	No effect				(112)
Gold fish	No effect	No effect (EFS)			(112)
Zebrafish	No effect				(35)
**Amphibians**					
Bullfrog	No effect				(36)
Japanese fire belly newt	No effect				(36)
**Reptiles**					Research not available
**Birds**					
Chicken	Contraction			Smooth muscle (crop and proventriculus)	(77)
		Potentiation of EFS		Enteric neurons (proventriculus)	(77)
Quail	No effect				(113)
					(41)
**Mammals**					
Human	No effect				(99)
			Contraction (Premature phase-III)		(114)
			Increase in gastric emptying		(115)
			Increase in gastrointestinal transit		(116)
Dog			No effect (motility and gastric emptying)		(117)
			Inhibition of phase-III	Decrease in motilin release	(49)
Rat	No effect	Potentiation of EFS		Enteric neurons	(99, 118)
			Contraction	Enteric neurons and vago-vagal reflex	(118, 119)
			Increase in phase-III		(26)
			Contraction (colon)	Defecation center	(120, 121)
Mouse	No effect	Potentiation of EFS		Enteric neurons	(24, 122)
			Increase in gastric emptying	Enteric neurons	(122)
			Increase in phase-III		(27)
Guinea-pig	No effect				(123–125)
		No effect (ganglion stimulant)			(124)
			Contraction (stomach)	Vago-vagal reflex	(123)
Rabbit	No effect				(126)
Asian house musk shrew	Contraction			Enteric neurons (presence of motilin required)	(19, 68)
			Contraction (phase-II), potentiation of phase-III	Enteric neurons and vago-vagal reflex	(20)

*EFS, Electrical field stimulation for excitation of enteric neurons*.

### Mammals

Effects of GHRL on GI motility were investigated using *in vivo* and *in vitro* experimental conditions in several mammals.

#### Rat and Mouse

Rats and mice are naturally deficient in the MLN system, as mentioned earlier (29), but they have the GHRL system instead [(11, 14), Figure 3]. GHRL bears GI motility regulating actions both *in vivo* and *in vitro* and increases gastric emptying (118, 119, 122, 127). The motility stimulation action of GHRL is caused by direct stimulation of the enteric neural pathway and capsaicin-sensitive vagal afferent neurons, which in turn stimulates parasympathetic efferent cholinergic neurons (118, 119, 122) (Figure 1, Table 2). Since GHS-R1a is not expressed in smooth muscles (99) and non-stimulated isolated gastric strips do not respond to GHRL (118, 122), direct action on smooth muscle cells is excluded as a stimulatory mechanism, different from the case of MLN. Interdigestive migrating complex (IMC)-like activity with short intervals (15–20 min) was also observed in the stomach of mice and rats (26, 27, 119), and GHRL enhances the appearance of the gastric IMC. Furthermore, a GHS-R1a antagonist, [D-Lys^3^]-GHRP-6, attenuates the frequency of IMC (26, 27), suggesting that GHRL serves as an alternative to MLN with regard to regulation of the gastric IMC in rats and mice.

In addition to the regulation of gastric motility, GHRL and its agonist applied into the lumbo-sacral spinal cord (region of the defecation control center) stimulated defecation of rats through activation of pelvic nerves and the connected enteric neurons (120). Intravenously injected GHRL failed to stimulate defecation (120), but a centrally acting GHRL receptor agonist, GSK894281, which is able to pass through the blood-brain barrier, was effective for stimulating defecation (128). GHRL-sensitive neurons are located in the lumbosacral defecation center and regulate defecation in rats (121) (Figure 1, Table 2). Therefore, in rodentia, GHRL is a GI motility regulator in both the stomach (digestion) and colon (defecation).

#### Guinea-Pig

Guinea-pig GHRL has recently been identified and demonstrated to locate in the mucosa of the stomach (125). As shown in Figure 3, GHRL in most mammals has a common ten-amino-acid sequence at the N-terminus (GSSFLSPEHQ), but in the guinea-pig, three amino acids are different at positions 2, 5, and 10 within the sequence (GASFRSPEHH). In spite of the unique amino acid sequence, guinea-pig GHRL is able to activate both guinea-pig and rat GHS-R1a with almost the same affinity (125). Guinea-pig GHRL increases food intake when injected intraperitoneally (125).

The GI motility-stimulating action of GHRL in the guinea-pig was investigated using both rat and guinea-pig GHRL under an anesthetized condition *in vivo* and isolated muscle strips *in vitro*. Rat GHRL increased gastric motility *in vivo* and its stimulatory action was decreased by atropine, hexamethonium, and capsaicin. Unacylated GHRL was not effective at inducing gastric contraction (123). In addition, non-stimulated and stimulated isolated GI strips were insensitive to rat GHRL and guinea-pig GHRL *in vitro*. ACh release from enteric neurons was not enhanced by rat GHRL (123–125). These results suggest that only the vago-vagal reflex pathway was involved in GHRL-induced gastric contractions and that GHRL mainly acted on the terminals of primary afferent neurons (123) (Figure 1, Table 2). Although the guinea-pig is a kind of rodentia, the actions of GHRL on enteric neurons reported in rats and mice have not been included in the mechanisms of GHRL-induced GI-stimulating actions. This may be due to very scant expression of GHS-R1a mRNA in the guinea-pig GI tract (125). Although the guinea-pig belongs to rodentia, IMC-like gastric motility observed in conscious mice and rats (26, 27) has never been reported. Therefore, the functional role of GHRL in regulation of the IMC has not been clarified.

#### Dog

Since dog indicates two patterns of GI motility depending on the feeding, the effect of GHRL has been investigated in the interdigestive state. GHRL intravenously applied at phase-I of the interdigestive state did not cause any mechanical changes and did not enhance gastric emptying in postprandial dogs, though GH-releasing activity was observed (117). However, interestingly, GHRL applied during phase-II and phase-III of the gastric IMC inhibited the appearance of phase-III and decreased plasma MLN level through activation of GHS-R1a (49). Regarding plasma GHRL concentrations, endogenous GHRL level changes in a cyclic pattern, i.e., the peak of plasma GHRL concentration is observed in the phase-I period, and the lowest GHRL concentration appears in the phase-III period. The cyclic changes of plasma GHRL are opposite to those of plasma MLN (49, 129). Consistent with this fact, exogenous GHRL decreases plasma MLN level and, conversely, exogenous MLN decreases plasma GHRL level. This indicates mutual regulation of MLN release and GHRL release for maintaining a regular interval of the IMC in dogs (49). There have been no *in vitro* studies regarding the action of GHRL in a dog GI tract. Gathering these results, GHRL might regulate dog GI motility indirectly through control of MLN release. The underlying detailed mechanisms of the cyclic changes of GHRL release in the dog remain to be determined.

#### Human

Different from the action of GHRL in the dog GI tract, it has been shown that GHRL stimulates gastric motility and accelerates gastric emptying in healthy human volunteers (114–116, 130, 131). Therefore, GHRL has been proposed as a target for therapeutics of GI motility disorders, such as delayed gastric emptying and postoperative ileus (132). Regarding regulation of the IMC in fasting periods, intravenously applied GHRL induces a premature gastric phase-III in phase-I quiescent periods without stimulating MLN release. This premature IMC was accompanied by a prolonged increase in gastric tonus (114). However, plasma GHRL concentrations did not change in a cyclic manner, different from the change in MLN concentrations during IMC cycles, and the magnitude of change in plasma GHRL levels was small compared with that of MLN (45). In addition, the concentration of GHRL that caused the premature IMC was considerably high compared with the normal endogenous GHRL level, suggesting that GHRL causes phase-III-like contraction at pharmacological doses, but it is not a physiological regulator of the IMC in humans, actually MLN acts the role for regulation of the IMC (45).

#### Rabbit

Isolated muscle strips from the rabbit stomach did not respond to GHRL even at a relatively high concentration (10 μM) (126). The results of an *in vivo* study have not been reported, but this kind of study is needed to examine the actions of GHRL on afferent vagus nerve terminals or the central nervous system.

#### House Musk Shrew

GHRL has been identified in the Asian house musk shrew (66) (Figure 3) and has been reported to stimulate gastric contraction in the latter half of phase-I and to enhance phase-II contractions. On the other hand, a GHS-R1a antagonist ([D-Lys^3^]-GHRP-6) suppressed the occurrence of spontaneous phase-II contractions and prolonged the time of occurrence of the peak of phase-III contraction. The pathway through the vagus nerve is essential for the GHRL-stimulating phase-II contraction (19, 20) (Figure 1, Table 2). Different contractile patterns in the interdigestive and digestive periods have been reported in the house musk shrew and interestingly, MLN has been shown to cooperate with GHRL in the initiation of phase-III-like gastric contractility (20, 54): in an *in vitro* study, treatment with GHRL alone did not cause any gastric contraction, but GHRL showed contractile activity in the presence of a very low concentration of MLN. On the other hand, pretreatment with GHRL enhanced MLN-induced gastric contraction, and a positive correlation between GHRL and MLN was also found in anesthetized animals *in vivo*. Therefore, GHRL is essential for the phase-II contraction, and coordination of MLN and GHRL is necessary to initiate the phase-III contraction of the stomach (19, 20). Intrinsic primary afferent sensory neurons that are located in the mucosa are necessary for the synergistic responses between GHRL and MLN, and GHRL enhances MLN-induced contraction by disinhibition of the GABA neuron-mediated tonic inhibition (68). Coordination between GHRL and MLN has so far been shown only in the house musk shrew, and this animal has been proposed as a suitable small laboratory animal for neuro-gastroenterological study of the gastric IMC and for study on the coordination of MLN and GHRL.

### Birds

#### Chicken

GHRL has been identified in chickens (Figure 3) and it has been shown that homologous GHRL stimulates GH release (108), but food intake is decreased by central and peripheral applications, different from the action observed in mammals (110, 133, 134). On the other hand, fasting increases plasma GHRL concentration as in mammals (135).

Since the opposite inhibitory effect on feeding regulation is observed in chickens, it would be interesting to examine the effect of GHRL on GI motility. Actually, GHRL acts on the chicken GI tract. In isolated GI muscle strips, chicken GHRL, but not rat GHRL, causes contraction of the upper intestine (such as the crop) and proventriculus and the lower intestine (such as the colon) but not of the middle intestine (such as the duodenum, jejunum and ileum). Interestingly, pharmacological analysis indicated different contractile mechanisms depending on the region: in the crop, GHRL acts on smooth muscle cells directly, and in the proventriculus, GHRL stimulates both smooth muscle cells and enteric neurons (77). Direct action of GHRL on smooth muscle cells is a characteristic of the chicken GI tract that has never been reported in mammals [Table 2, (136)]. The region-dependent contractile responses correlate to the expression level of GHS-R1a mRNAs, indicating that the different degrees of responsiveness of the GI tract to GHRL are due to the heterogeneous expression of GHS-R1a (76). The region-dependency of the GHRL-induced contraction is in contrast to that of the MLN-induced contraction (77): in the crop, proventriculus and colon, which are sensitive to GHRL, MLN showed a weak effect, and in the middle intestine, MLN caused strong contraction, whereas GHRL caused only a small contraction. This has never been observed in mammals.

Ontogenic and developmental changes in morphology and function of the GI tract in the chicken have been reported. Since GHRL stimulates GH release and regulates energy homeostasis, we hypothesized that the degree of GHRL-induced contraction may change in growing chickens (76). In fact, GHRL-induced responses in the proventriculus decreased depending on age from day 0 to day 100 after hatching, and the decreased contraction was correlated with reduction of GHS-R1a mRNA expression. However, age-dependent decreases in contraction and GHS-R1a mRNA expression did not occur in the crop. Therefore, the crop is a physiological target for GHRL in the chicken. A negative significant correlation between plasma GHRL level and GHS-R1a mRNA expression level in the proventriculus suggests down-regulation of GHS-R1a mRNA expression by increased plasma GHRL concentration to maintain the GHRL-induced response within a certain level (76, 137). The detailed unique mechanism of down-regulation of GHS-R1a mRNA in the proventriculus is unknown. However, GHS-R1a is mainly expressed on smooth muscle cells in the crop, whereas it is expressed on both smooth muscle cells and neural components in the proventriculus (77, 137). These different distributions of neural and muscular GHS-R1a may be responsible for the different aging-dependent changes: neural GHS-R1a may be easily down-regulated by increased endogenous GHRL. Similarly, contraction of the proventriculus induced by MLN, which involves both myogenic and neural mechanisms, decreases depending on age, but such a decrease does not occur in the ileum, where MLN acts on muscle receptors (76). In addition, contractions induced by carbachol and serotonin, which act on myogenic muscarinic and serotonin receptors, do not decrease with aging (76). Therefore, the age-dependent decrease in the responses to GHRL is due to the decreased neural GHS-R1a in enteric neurons of the proventriculus. In contrast to those *in vitro* studies, no *in vivo* study focusing on the regulation of GI motility by GHRL has been performed. The physiological significance of GHRL has not been fully clarified.

#### Quail

Quail GHRL and GHS-R1a have been identified (103, 113, 138). Intraperitoneal (ip) injection of a small dose of GHRL, but not intracerebroventriclar (icv) injection of small doses, stimulates food intake, whereas large doses of GHRL, injected as both ip and icv, inhibit feeding as seen in chickens (139). Despite the different actions of GHRL on food intake, the pattern and degree of GHS-R1a mRNA expression in the GI tract are similar to those in chickens: i.e., the expression level of GHS-R1a mRNA is high both in the upper regions (esophagus, crop, and proventriculus) and lower regions (colon) of the GI tract and relatively low in regions of the middle intestine (duodenum, jejunum, and ileum) (113). In this condition, chicken GHRL-induced contraction was very weak in all GI regions of the quail despite the fact that similar levels of GHS-R1a mRNA were expressed (113). Ineffectiveness of chicken GHRL in the quail proventriculus and duodenum has also been reported (41). Such a discrepancy between GHS-R mRNA expression level and contractile response has been reported in humans: substantial amounts of GHS-R mRNA and its protein are expressed in the stomach and colon (140), but GHRL did not cause mechanical responses or modify neural contractions (99). The underlying mechanisms are still not known, but there are some possibilities: (i) GHS-R1a mRNA has not been translated in GHS-R1a protein and (ii) most of the GHS-R1a is not involved in intestinal contraction and is linked to other intestinal functions as has been suggested in fish (141, 142). Since GHRL actions that we observed in the chicken and quail are quite different, it is necessary to examine the effects in other bird species in order to clarify the common action of GHRL in the avian GI tract. In addition, *in vivo* experiments are needed because GHRL regulates the GI tract by activation of the vago-vagal reflex and/or by activation of the CNS.

### Reptiles

A GHRL peptide has been identified in the red-eared slider turtle (*Trachemys scripta elegans*) [Figure 3, (143)] and the partial sequence of squamata has been deposited in the NCBI database. However, nothing is known about its physiological roles such as GH release, food intake and GI motor functions in reptiles.

### Amphibians

A GHRL peptide has been identified and its amino acid sequence has been determined in several species of amphibians [Figure 3, (84, 103, 107, 144, 145)]. GH-releasing activity has been demonstrated (107) and it has been shown that the endogenous GHRL level is increased by fasting (144) and GHRL stimulated food intake in the bullfrog larvae (146), indicating involvement in energy homeostasis. However, neither bullfrog GHRL nor rat GHRL caused contraction of the stomach and intestinal muscle strips, despite the fact that other stimulants, such as substance P and a muscarinic agonist, caused marked contraction (36). Similar to the bullfrog, GI strips from Japanese fire belly newts were also insensitive to newt GHRL and rat GHRL *in vitro* (36). The expression level of GHS-R1a in the bullfrog is comparable to that in chickens (36, 113), and this is the same as the case of the quail GI tract. GHS-R1a mRNA was expressed more predominantly in the mucosa than in the smooth muscle in the bullfrog intestine (36), indicating the possibility that GHRL in the bullfrog does not regulate GI motility but regulates mucosal functions, such as absorption of nutrients and secretion. On the other hand, the expression level of GHS-R1a mRNA in the GI tract of the Japanese fire belly newt was only about 10% of that in the GI tract of the bullfrog, and GHRL did not affect GI motility in the newts. A functional approach using isolated strips failed to demonstrate GHRL function in GI motility in an *in vitro* study, and it is necessary to investigate functions such as gastric emptying, intestinal transient and GI motility in an *in vivo* study.

### Teleost Fish

The gene and peptide structures of GHRL have been identified in various fish species [Figure 3, reviewed by Kaiya et al. (84)]. As in mammals, GHRL mRNA was mainly expressed in the stomach of fish and in the intestine in stomach-less fish, such as goldfish and zebrafish. GHRL regulates energy balance through its actions on release of pituitary hormones, food intake, and lipid metabolism in fish (103, 110, 147).

*In vitro* experiments on the GI motility stimulation actions of GHRL have been performed in several fish. In the isolated rainbow trout (*Oncorhynchus mykiss*), stomach and intestinal strips, two types of rainbow GHRL [20 and 23 amino acids, (148)] did not cause any mechanical changes (112). The goldfish (*Carassius auratus*) is a stomach-less fish and has a thick intestine called the intestinal bulb instead of the stomach. Two types of goldfish GHRL, with different fatty acid modifications of Ser-3 (octanoyl and decanoyl forms) (149), and rat GHRL did not cause any mechanical changes in a preparation from the intestinal bulb. Stimulation of enteric nerves caused contraction by excitation of both cholinergic and non-cholinergic nerves, but both the octanoyl and decanoyl forms of goldfish GHRL applied during the stimulation did not modify the neural responses (112) (Table 2). These results indicate that GHRL does not play a crucial role for GI motor function in the rainbow trout and goldfish, being different from the results for chickens, mice and rats.

It is thought that GI motor functions might be markedly different in land-dwelling creatures and underwater creatures because of different kinds of food, living temperature and surrounding osmotic environment. Therefore, the lack of GI motility regulatory actions of GHRL and MLN observed in the rainbow trout, zebrafish, and goldfish might reflect these differences. However, little has been reported about the difference in the motor functions of GI between fish and mammals.

## Conclusion

This review focused on control of GI motility by MLN and GHRL in vertebrates. Both peptides are thought to originate from the same ancestral gene, and they are predominantly produced in and released from the mucosa of the GI tract and act on MLN-R and GHS-R1a. The two peptides have various functions, and their target is not only the GI tract. The GI motility-stimulating actions are different from animal species and seem to be reflected during the vertebrate evolution process (Table 3).

**Table 3:** Summary of the ghrelin and motilin systems in various vertebrates and their actions on gastrointestinal motility.

	**Motilin**	**Motilin receptor**	**GI motility-stimulating action**	**Ghrelin**	**GHS-R1a**	**GI motility-stimulating action**
Fish	○	○	×	○	○	×
Amphibians	ND	○	Δ (only bullfrog)	○	○	×
Reptiles	○	○	NE	○	○	NE
Birds	○	○	○	○	○	○ (only chicken)
Mammals	○ (except rodenita)	○ (except rodentia)	○ (except rodentia)	○	○	○

*○, presence or determined; Δ, very weak and doubtful; ×, No effect*.

*ND, Not determined; NE, Not examined*.

GHRL and MLN are present in fish, and the structure (amino acid sequence) of the identified MLN (called MLN-LP) is slightly different from that of avian and mammalian MLN. Their functional receptors are expressed in the GI tract, but neither of the peptides is involved in regulation of GI motility. In amphibians, neither MLN nor GHRL shows a remarkable effect on GI motility despite the considerable presence of receptors in the GI tract. Depending on species, MLN may have begun to control GI motility partially as observed in the bullfrog, but not by GHRL. Nothing is known about GI motility stimulating actions of the peptides in reptiles, but the structure of the MLN-like peptide changes into an avian and mammalian type in some species. In avian species, although expression levels of receptors are sufficient to function, species where GHRL and MLN stimulate GI contraction or not are more apparent, and a region-dependent control of GI motility by GHRL (upper and lower intestines) and MLN (middle intestine) has begun to observe. Only after this evolutional process, the ligand-receptor-GI motility regulation system begins to link to causing contraction, and the mechanism varies depending on the part of the GI tract. In chickens, it has been demonstrated that GHS-R1a is expressed on both smooth muscle cells and enteric neurons and that age-dependent down-regulation of neural GHS-R1a occurs. Furthermore, when the evolutional stage is reached in mammals, the diversity of stimulatory mechanisms is further increasing. In rats and mice, the MLN system is lost and GI contraction is controlled only by the GHRL system. The guinea-pig is losing the MLN system at the molecular level and is also losing regulation of GI motility by local actions of GHRL. Moreover, in the house musk shrew, GHRL and MLN show a cooperative effect in regulation of GI motility, and the action was specialized in the regulation of phase-III contraction. GHRL is a physiological regulator of the IMC in rodentia and the house musk shrew, and MLN regulates the gastric IMC in a fasting state in humans and dogs and translates the hunger signal from the periphery to the brain (Table 3).

In this way, while MLN and GHRL are hormones produced in the GI tract, they are not initially involved in the regulation of GI motility. However, it seems to act on the regulation of GI motility more when nearer the region of producing cells (GI tract), with animal evolution (Table 3). If that is so, what is the action of MLN or GHRL to begin with? Why did they not act in the GI tract despite being produced there? Why did they come to affect GI motility? It is interesting to anticipate it from the change of action when considering vertebrate evolution.

## Author Contributions

TK and HK contributed almost same degree in completing the review.

### Conflict of Interest Statement

The authors declare that the research was conducted in the absence of any commercial or financial relationships that could be construed as a potential conflict of interest.
